# Minimal Gluten Exposure Alters Urinary Volatile Organic Compounds in Stable Coeliac Disease

**DOI:** 10.3390/s22031290

**Published:** 2022-02-08

**Authors:** Michael McFarlane, Ramesh P. Arasaradnam, Beryl Reed, Emma Daulton, Alfian Wicaksono, Heena Tyagi, James A. Covington, Chuka Nwokolo

**Affiliations:** 1 Department of Gastroenterology, University Hospitals Coventry and Warwickshire, Clifford Bridge Road, Coventry CV2 2DX, UK; ramesh.arasaradnam@uhcw.nhs.uk (R.P.A.); chuka.nwokolo@uhcw.nhs.uk (C.N.); 2 Faculty of Health Science, University of Coventry, Coventry CV2 2DX, UK; 3 Department of Dietetics, University Hospitals Coventry and Warwickshire, Clifford Bridge Road, Coventry CV2 2DX, UK; beryl.reed@btopenworld.com; 4 School of Engineering, University of Warwick, Coventry CV4 7AL, UK; e.daulton@warwick.ac.uk (E.D.); a.wicaksono@warwick.ac.uk (A.W.); h.tyagi@warwick.ac.uk (H.T.); j.a.covington@warwick.ac.uk (J.A.C.)

**Keywords:** coeliac disease, VOCs, FAIMS, gluten challenge, gluten free diet

## Abstract

Coeliac disease (CD) patients are distinguishable from healthy individuals via urinary volatile organic compounds (VOCs) analysis. We exposed 20 stable CD patients on gluten-free diet (GFDs) to a 14-day, 3 g/day gluten challenge (GCh), and assessed urinary VOC changes. A control cohort of 20 patients continued on GFD. Urine samples from Days 0, 7, 14, 28 and 56 were analysed using Lonestar FAIMS and Markes Gas Chromatography–Time of Flight–Mass Spectrometer (GC-TOF-MS). VOC signatures on D (day) 7–56 were compared with D0. Statistical analysis was performed using R. In GCh patients, FAIMS revealed significant VOC differences for all time points compared to D0. GC-TOF-MS revealed significant changes at D7 and D14 only. In control samples, FAIMS revealed significant differences at D7 only. GC-TOF-MS detected no significant differences. Chemical analysis via GC-MS-TOF revealed 12 chemicals with significantly altered intensities at D7 vs. D0 for GCh patients. The alterations persisted for six chemicals at D14 and one (N-methyltaurine) remained altered after D14. This low-dose, short-duration challenge was well tolerated. FAIMS and GC-TOF-MS detected VOC signature changes in CD patients when undergoing a minimal GCh. These findings suggest urinary VOCs could have a role in monitoring dietary compliance in CD patients.

## 1. Introduction

Coeliac disease (CD) is a T-cell mediated gluten sensitive enteropathy, affecting approximately 1% of the global population [1]. CD is diagnosed by histopathological examination of small bowel biopsies from the second part of the duodenum. This is often preceded by serological investigations, such as anti-tissue transglutaminase (TTG) antibodies, anti-gliadin and anti-endomysial antibodies [2,3]. The value of these serological tests in monitoring response to a gluten-free diet (GFD) is uncertain [4,5,6]. In patients with immunoglobulin A (IgA) deficiency, IgA antibodies can be undetectable, leading to false-negative results [6,7].

Tissue sampling for diagnosis of CD requires the consumption of a gluten-inclusive diet for at least 8 weeks prior to biopsy [8], but Leffler et al. [9] demonstrated that duodenal mucosal changes are detectable 2 weeks after starting a gluten challenge (GCh) of >3 g of gluten/day. Among patients who developed symptoms, these changes had returned to baseline 2 weeks after the cessation of the challenge. The authors concluded that ingestion of 3 g daily of gluten was sufficient to induce the serological and histological changes of coeliac disease. Subsequent studies of GCh have shown that doses as high as 10 g/day are needed for histological changes, but that 3 g/day is enough to bring on symptoms in a 2-week GCh [10], and that a GCh of 5.7 g/day was enough to induce human lymphocyte Antigen (HLA) subtype DQ changes, but not histological changes [11].

The detection of specific patterns of volatile organic compounds (VOCs) using multiple technologies, including electronic noses (E-noses) and related technology, in urine, breath, sweat and faeces has been applied to the non-invasive diagnosis of gastrointestinal conditions, including inflammatory bowel disease, bile acid malabsorption, colorectal cancer and radiation-induced GI side effects [12,13,14,15,16,17].

In an earlier study we used field asymmetric ion mobility spectrometry (FAIMS) to distinguish CD patients from diarrhoea predominant irritable bowel syndrome (D-IBS) via urinary VOC analysis with a sensitivity and specificity of 85% [18]. FAIMS achieves the separation of VOCs as a result of the different molecular mobilities of chemicals in response to cycling of high and low electrical fields. This study also identified, via GCMS (gas chromatography and mass spectrometry), a unique chemical peak at 4’67 found only in CD, and not D-IBS, which correlated with the compound 1, 3, 5, 7 cyclo octatetraene [18].

A study of faecal VOCs in refractory CD patients and CD patients on a GFD using gas chromatography–ion mobility spectrometry (GC-IMS) technology, found that refractory CD could be differentiated from CD on a GFD with an area under the curve (AUC) of 0.91 [19]. These studies suggest that VOCs could provide a non-invasive method for screening and monitoring GFD compliance in CD.

Our study aimed to analyse urinary VOCs in stable CD patients on a GFD undergoing a 14-day GCh of biscuits containing 3 g of gluten/day. The overall purpose of these experiments was to determine whether the urinary VOC signatures of stable CD patients changed in response to GCh, and whether it reverted to the pre-challenge signatures after the cessation of the challenge. To achieve this, we used a combination of a FAIMS technique (E-nose family), which can be deployed as a point of care test and has previously shown applicability to inflammatory conditions, with GC-TOF-MS (gas chromatography-time of flight-mass spectrometry) to identify specific biomarkers resulting from the challenge, and to determine whether the two technologies gave comparable results.

## 2. Materials and Methods

### 2.1. Patients

Forty stable, long-term CD patients, diagnosis confirmed histologically, with self-reported compliance with GFD and a normal TTG in the preceding 3 months, were recruited. They were asked to either receive a gluten challenge (GCh patients, *n* = 20) or to continue with their GFD (non-GC patients, *n* = 20). The non-GCh patients acted as a control group.

Exclusion criteria were non-coeliac organic GI disease, history of non-gluten food intolerances/allergies and treatment with corticosteroids or immunosuppressant drugs in the preceding 3 months.

### 2.2. Study Design

The GC patients received 3 cracker biscuits (Jacob’s Cream Crackers ©, Jacobs, UK), daily in addition to their regular GFD for 14 days. Each cracker biscuit contains 1 g of gluten [20] so each GCh patient ingested 3 g of gluten daily for 14 days. The non-GCh patients remained on a GFD.

Urine samples were collected from both groups on day 0 (pre-GCh), and on days 7 and 14 (end of GCh), and then on days 28 and 56. Five millilitre blood samples for serum TTG concentration were also collected on Days 0, 14 and 56. Patients completed a symptom diary and were allowed to decrease or stop the GCh if symptoms became intolerable. Analyses were done on an intention-to-treat basis.

Table 1 shows a study timeline summary.

### 2.3. Sample Collection and Storage

Patients provided 20 mL samples of early morning urine, which were voided into standard universal Sterilin specimen containers (Newport, UK) and stored at −80 °C within 24 h of voiding. Urinary VOCs have been shown to be stable at room temperature during this period [19]. These samples were then shipped to the University of Warwick for testing, where they were left in a laboratory fridge at 4 °C overnight to defrost prior to analysis.

### 2.4. Sample Analysis

#### 2.4.1. FAIMS

FAIMS is an approach where VOCs can be detected by measuring their mobilities in high and low electric fields. Molecules are ionized and then passed between two plates to which an asynchronous voltage is applied. This alters the path of the ion, which is compensated for by applying a DC voltage and allowing the ion to be detected. Thus, the mobility measurement is a function of the amplitude of the high/low electric fields and the compensation voltage.

For the FAIMS analysis, a commercial instrument was used (Lonestar, Owlstone Medical, Cambridge, UK). This was attached to an ATLAS sampling system (Owlstone Medical, Cambridge, UK). In use, a 5 mL sample aliquot was placed into a 10 mL vial. The sample was then placed in the ATLAS sampling system and heated to 40 ± 0.1 °C for 10 min. The flow rate over the sample was set to 200 mL/min using clean/dry air as the carrier gas. The sample air was further diluted with zero air to create a total flow of 2 L/min. The Lonestar was scanned from 0 to 99% dispersion field in 51 steps and +6 V to −6 V compensation voltage in 512 steps, with both positive and negative ions used. This created a output file per sample of 52,224 data points. Each sample was scanned three times and air blanks were used in between samples to ensure that there was no carry over. Furthermore, our standard quality control checks were performed before starting the experiment to ensure the results were reliable.

#### 2.4.2. GC-TOF-MS

GC-TOF-MS is a hybrid technology composed of a gas chromatography column in sequence with a time-of-flight mass spectrometer. This allows the separation of chemical compounds before they are applied to the mass spectrometer and provides better separation for analysis.

For GC-TOF-MS, 5 mL of defrosted urine sample was aliquotted into a 20 mL borosilicate vial (Thames Restek, High Wycombe, UK) containing a Markes Bio-Monitoring sorbent tube (C2-AAXX-5149, Markes Intl., Llantrisant, UK), and sealed with a crimp cap. The samples then heated for 60 min at 40 °C to absorb the urine onto the sorbent tube for analysis.

Once complete, the sorbent tubes were sealed using Markes DiffLok caps and loaded into a Markes Unity and Ultra Thermal Desorber auto-sampler system for analysis using a Markes BenchTOF-HD using a Rxi-624SilMS 20 m 0.18 mm ID, 1.0 um column (Thames Restek 13865).

The TOF-HD analysed masses between 35–250 atu (atomic mass units) for a total run time of 25 min. The filament voltage was set to 1.7 V, and the filament delay to 10 s. The sample transfer line was set to 250 °C, as was the ion source temperature, with ionisation at −70 V. During the analysis dynamic background compensation (DBS) was automatically applied by the software, which integrates and deconvolutes the peaks present in the sample. The integration settings were as follows: global height reject, 10,000; global width reject, 0.001; baseline threshold, 3; and global area reject, 10,000. The peaks were then identified using the NIST list, with a forward and reverse match with a minimum match factor of 450. These settings were used based on previous clinical studies involving urine samples [21] In both the case of FAIMS and GC-TOF-MS, all the samples were tested in a single batch to remove batch to batch variations.

#### 2.4.3. TTG

Blood samples were collected in standard 5 mL biochemistry bottles and then analysed using the EliA Celikey IgA assay (Thermofisher, Waltham, MA, USA), as per the manufacturer’s guidelines. The normal range of the assay is <5 kU/L.

### 2.5. Statistical Methods

Although no formal calculation of sample size was performed, previous VOC studies performed by our group have shown that sample and control groups of 15–20 patients are able to achieve statistically significant results [14,15,16,17,18].

All statistical analyses were performed using R version 3.4.1. For the Lonestar analysis, all the samples were exported to text file and the second run used for analysis. From previous studies, we have found that the second run provides the most reliable information content. The positive and negative scans were combined into a single matrix and a threshold applied to remove the background information. In this case, any values below the threshold were set to zero and were set at twice the average background level, with the same value used for all the files. Once completed, a number of comparisons were undertaken comparing different days to day 0. For each comparison, a 10-fold cross validation was used, where the data are divided into 10 equal groups, with 9 groups using to create a model that was applied to the 10th group. This was repeated 10 times until all the samples were within a test group. For the training group, a rank-sum test was performed on each data point, across all the samples, to identify those that held discriminatory information. The 20 data points with the lowest *p*-value were then used to create a model. A sparse logistic regression model was applied from the resultant probabilities; statistical parameters including ROC (receiver operator characteristics) curves, sensitivity, and specificity, area under the curve, negative predictive value, positive predictive value and *p*-value were calculated.

For the GC-TOF-MS, a similar 10-fold cross validation process was undertaken. Here, the identified chemicals were used as features and the abundance as its value. In addition, due to the smaller dimensionality of the data at this point, no feature extraction approach was required and the models were built directly on the output values. Analysis of the chemical composition and differences between each time point and Day 0 was performed using a paired *T*-test analysis with a *p* value of <0.05 to identify significantly different chemicals between each time point.

## 3. Results

There were no significant differences in age and sex when the GCh and non-GCh patients were compared. However, one of 20 GCh patients returned no samples. Of the remaining 19 patients, 18 ingested the crackers as per protocol and one patient reduced their crackers intake in the last 3 days by 25%. This patient was included in the analyses. These 19 patients returned their urine samples and symptom diaries.

Three of 20 non-GCh patients returned no samples and 17 of 20 returned their urine samples and symptom diaries. Table 2 shows the demographic data.

### 3.1. Urinary VOCs

#### 3.1.1. FAIMS

The pre-GCh urine sample signatures on Day 0 were compared to the signatures of the samples on Days 7, 14, 28 and 56 using the sparse logistic regression model.

Among the GCh patients (*n* = 19), when compared to Day 0, the signatures were significantly different on days 7, 14, 28 and 56. The AUC decreased from 0.92 (0.85–0.99) on Day 7, to 0.80 (0.64–0.95) on Day 14, to 0.71 (0.53–0.89) on Day 28 and 0.77 (0.62–0.93) on Day 56. *P* values for the four time points were <0.001, <0.001, 0.01 and 0.002, respectively.

**Figure 1:**
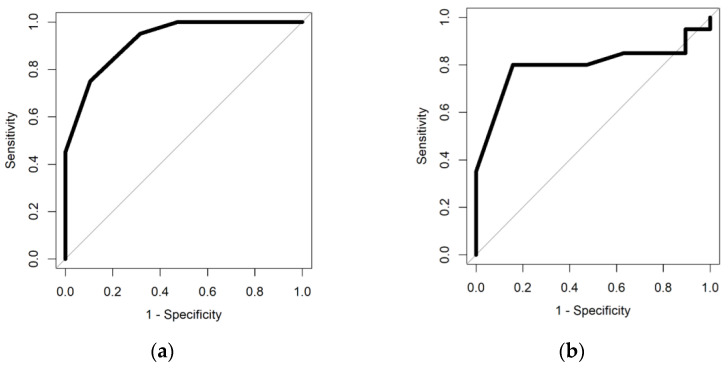
ROC curves for GCh patients using FAIMS Day 0 vs. Day 7 (**a**) and Day 14 (**b**).

Figure 1a,b show the FAIMS ROC curves, whilst Table 3 shows the full results of the FAIMS analysis.

Among the non-GCh patients, when compared to Day 0, there was a significant difference in VOC signatures on Day 7 (*p* = 0.002), but not on Days 14, 28 and 56.

#### 3.1.2. GC-TOF-MS

The pre-GCh urine sample signatures on Day 0 were compared to the signatures of the samples on Days 7, 14, 28 and 56 using a Gaussian process.

Among the GCh patients (*n* = 19), compared to Day 0, signatures were significantly different on Days 7 and 14. When comparing Day 0 with Day 7 and Day 0 with Day 14, the sensitivities were 0.65 (range 0.44–0.83) and 0.71 (range 0.52–0.88), the specificities were 0.64 (range 0.42–0.85) and 0.69 (range 0.45–0.90) and the *p* values were 0.02 and 0.05, respectively. There was no difference in VOC signatures when Days 28 and 56 were compared to day 0.

Among the non-GCh patients, there was no significant difference in VOC signatures when Days 7, 14, 28, and 56 were compared to Day 0.

**Figure 2:**
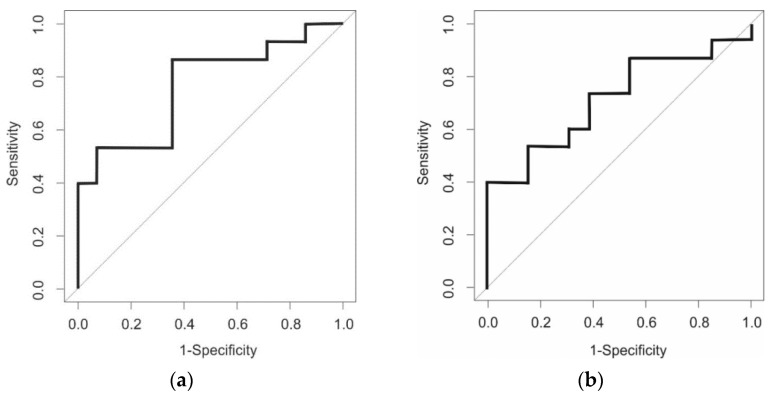
ROC curves for GCh patients using GC-TOF-MS Day 0 v Day 7 (**a**) & Day 14 (**b**).

Figure 2a,b show the GC-TOF-MS ROC curves, whilst Table 4 shows the results.

### 3.2. Chemical Analysis

The T test analysis of the chemical composition of the VOC signatures derived from the GC-TOF-MS data revealed 12 chemicals with significant alterations among the GCh patients between Day 0 and Day 7. There was a mean 52.6% decrease in chemical concentrations. Six of these chemicals remained altered on Day 14 and one (N-methyltaurine), remained altered until Day 56. Among the non-GCh patients, no consistent chemical alterations was detected throughout the study period.

**Figure 3:**
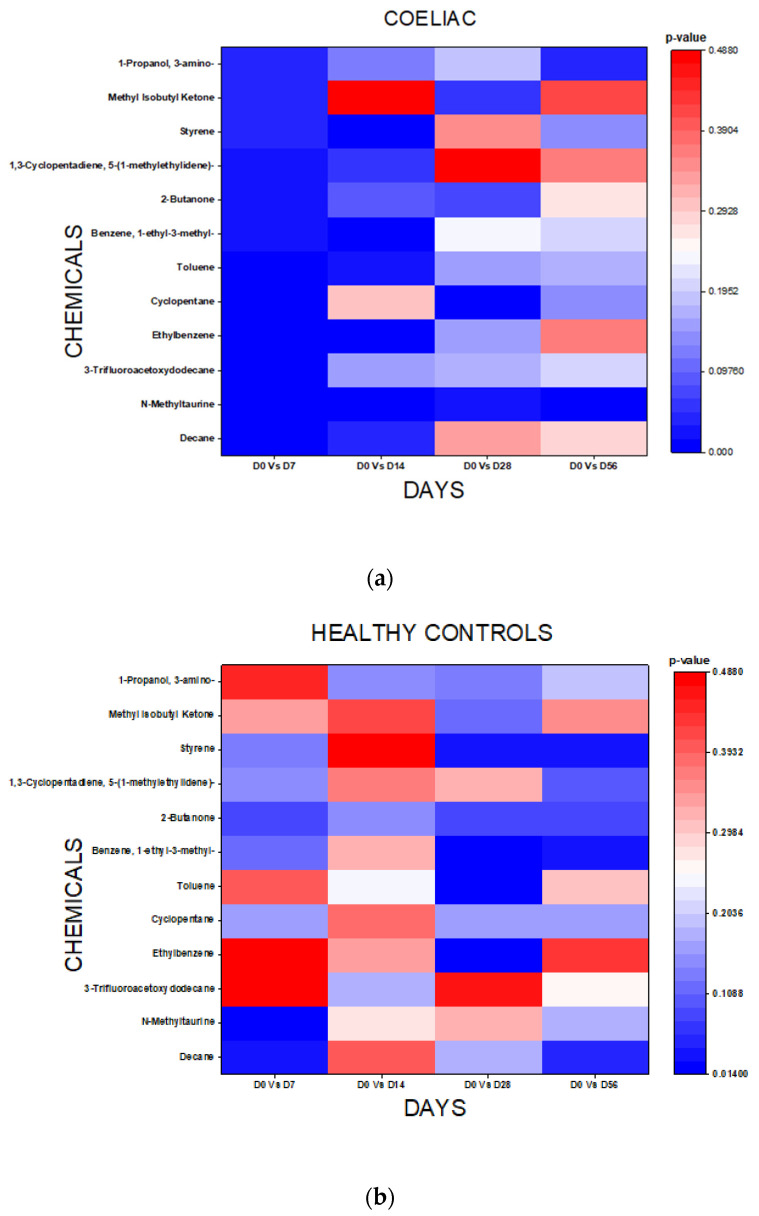
Heat map plots of chemicals and *p* values for GCh (**a**) and control groups (**b**).

Figure 3a,b show the heat map plots of the *p* values from the analysis.

### 3.3. Symptom Diaries

On review of the symptom diaries for the GCh patients, 73.5% (14/19) reported bloating, lethargy, loose stools and diarrhea that persisted during the challenge period. These symptoms were not sufficiently disabling to cause a reduction in gluten consumption for 18 out of 19 subjects; none terminated the challenge. Their symptoms resolved within a mean of 4.1 (range of 0–41) days post-challenge.

The non-GCh patients reported fewer symptoms than the GCh patients, with 53.0% (9/17) reporting brief episodes of bloating or loose stools; none for more than 24 h duration, and none coinciding with the significant VOC difference seen on Day 7.

### 3.4. TTG

Among the GCh patients, mean TTG antibody levels were 2.43 U/mL, 3.6 U/mL and 3.58 U/mL, at Days 0, 14 and 56, respectively. The increase was non-significant. In the non-GCh patients, the mean TTG antibody was unchanged at 1.17 U/mL, 1.20 U/mL and 1.10 U/mL on Days 0, 14 and 56, respectively. These results are shown in Table 5.

## 4. Discussion

The ingestion of cracker biscuits was associated with increased gastrointestinal symptoms among the GCh patients, although only 1 of 19 patients reduced their cracker biscuit intake, and only for the last 3 days of the challenge. There were only mild, brief non-specific symptoms among the non-GC patients, suggesting that the GCh patients’ symptoms could be attributed to the ingested gluten.

We used FAIMS and GC-TOF-MS to analyse identical urine samples and see if changes could be detected by both methods to lend authenticity to the observations. The GC-TOF-MS analysis allowed the identification of specific chemicals.

Both techniques identified a change in VOC signatures on Days 7 and 14 among patients who underwent the challenge. This change was no longer apparent by Day 28 when assessed by GC-TOF-MS, but was still detectable up to the final study time point (Day 56) by FAIMS analysis. There was no accompanying significant increase in TTG antibodies during the challenge, suggesting that VOC signature changes occur prior to any changes in this biochemical marker. If this is due to gluten, the presence of changes in VOCs by day 7 of the challenge suggests that this method of assessment is highly sensitive to gluten ingestion in stable CD patients. This could indicate a potential role for the technology in non-invasive CD monitoring and follows an earlier study to demonstrate faecal VOC changes in refractory CD, compared to those in remission on GFDs [17].

Urine samples were snap-frozen on receipt of samples, but previous studies suggest VOCs can still be assessed for up to 72 h after voiding [22] at room temperature, making this body fluid a potential candidate for the assessment of samples sent by post.

The underlying reasons behind why ingestion of three crackers biscuits daily might alter VOCs signatures in patients with coeliac disease stable on a GFD is uncertain. It could be a non-specific effect unrelated to the small amount of gluten contained in these biscuits. However, the accompanying symptomatic response in the GCh patients supports the view that gluten could be the cause of the changes noted in VOC signatures in urine.

FAIMS method detected ongoing VOC alterations after the GC ended, up until Day 56. The strength of signal decreases as shown by the decreasing AUC, sensitivity and specificity. FAIMS might detect changes caused by gluten ingestion that are more persistent. In contrast, the GC-TOF-MS methodology seems to detect changes occurring during the period when gluten is still being ingested. The underlying mechanism behind these changes could be an alteration of the GI microbiome or small bowel duodenal inflammation caused by gluten, although the latter is more plausible given the promptness of the changes noted. An increase in small intestinal permeability associated with gluten-induced inflammation might lead to luminal substances entering the circulation and subsequently excreted in urine. The discrepancy between the two methodologies could be that while the FAIMS detects the effects of gluten-induced mucosal inflammation, the GC-TOF-MS detects a component of ingested gluten. Gas chromatography-time of flight-mass spectrometry (GC-TOF-MS) hybrid technology is able to detect masses between 35–250 atomic mass units, but is novel in regard to its intended purpose in this study. Previous studies have used field asymmetric ion mobility spectrometry (FAIMS) to distinguish between patients with refractory CD from those that respond to GFD.

Chemical analysis using the GC-TOF-MS VOC assay showed among the GC patients that the abundance of 12 chemicals decreased by an average of 52% by Day 7. Only six chemicals were still decreased on Day 14. One chemical, N-methyltaurine, was decreased at all time points. N-Methyltaurine is an organosulphonic acid and has been previously identified as a common untargeted urinary metabolite [23]. It is unclear why it remains decreased across all time points for the GCh patients but only Day 7 for the non GCh patients. Other chemicals identified, such as the hydrocarbons decane and toluene, have been shown to be decreased in the saliva of children with CD compared to healthy controls [24] and in the faeces of treated CD compared to untreated CD [25]. The ketone 2-butanone has been shown to be decreased in saliva and urine [25,26] of children with CD.

Half of the observed chemical changes had normalised by the end of the GCh, with N-methyltaurine still abnormal at Day 28. By Day 56, there were no residual chemical changes. These changes may represent either the presence of gluten in the diet or alterations in the patients’ metabolic processes induced by small intestinal inflammation and/or alterations in the gut microbiome. Further studies with the corresponding microbiome analysis and small intestinal histological sampling would be needed to fully elucidate the underlying processes involved.

In non-GC patients, urinary VOCs remained unchanged for the duration of the study using GC-TOF-MS and were significantly different for one time point using FAIMS; this may represent background dietary gluten contamination. Future studies could consider the use of urinary gluten immunogenic peptide (GIP) to determine whether accidental gluten exposure has taken place in the control cohorts.

There are a number of limitations of this study. First, the FAIMS system and the GC-TOF-MS may not detect the same chemicals. The FAIMS system is undertaking direct headspace sampling, whilst the GC-TOF-MS process uses a pre-concentration stage. The tube used on the GC-TOF-MS was designed for measuring chemicals C4 and above; therefore, chemicals below C4 are unlikely to be detected. In addition, it was not possible to identify the specific chemicals detected by the FAIMS system, but the improved results for FAIMS may indicate that the chemicals being detected have a high proton affinity or are smaller and were not collected onto the tube. The advantages of FAIMS are that the technology is cheaper than GC-TOF-MS and that it can be used away from a lab setting and does not require specialised gases to operate. This makes it a potentially clinically useful approach in the future. A second limitation is that we did not undertake chemical confirmation of those identified in this study nor performed calibration for concentration. However, this is common with pilot studies of this nature and, if such a test is to be used in the future, further work will be necessary to confirm and quantify these chemicals.

Further limitations related to study design include the levels of gluten consumed for the challenge. Whilst sufficient to induce symptoms in our cohort, and equivalent doses have previously shown histological changes in the small bowel [9], it is worth considering that most dietary gluten contamination is likely to be at doses far lower than equivalent to three cracker biscuits (3 g) per day; therefore, further studies of lower dose ranges would be needed to ascertain whether consumption at lower levels is detectable in altered VOC signatures. It would also be of value to sample more frequent time points, particularly in the early stages of the GCh, to determine at what point the VOC changes become apparent, and whether this coincides with the acute cytokine changes which that been detected within 2–4 h of gluten exposure in CD patients [10,27]. Again, due to the pilot nature of this study, these considerations have only become apparent on analysis of the results.

## 5. Conclusions

This study provides further evidence of the potential of a role for urinary VOC analysis in patients with CD, and demonstrates the first use of GC-TOF-MS for this purpose. Earlier studies have demonstrated that FAIMS could distinguish CD from healthy controls and D-IBS. Here, we demonstrate that a 14-day GCh results in alterations in VOC signature changes in the urine of stable CD patients using both FAIMS and GC-TOF-MS. These changes appeared by Day 7 and resolved after completion of the GCh as shown by GC-TOF-MS technology. Our findings suggest a role for urinary VOC assessment in monitoring GFD compliance in CD patients where the inadvertent ingestion of small amounts of gluten can be a problem.

## Figures and Tables

**Table 1:** Timeline of study for GCh and non-GCh patients.

Day	Gluten Challenge (Cohort 1)	Controls (Cohort 2)
Day 0	Provide urine and Blood (TTG) sample	Provide urine and Blood (TTG) sample
Day 1	Begin GCh	Remain on GFD
Day 7	Provide urine sample	Provide urine sample
Day 14	Complete GCh Provide urine and Blood (TTG) sample	Provide urine and Blood (TTG) sample
Day 15	Return to GFD	-
Day 28	Provide urine sample	Provide urine sample
Day 56	Provide urine and Blood (TTG) sample	Provide urine and Blood (TTG) sample
Day 57	End of study	End of study

**Table 2:** Demographic data for all subjects.

Demographic	GCh Patients (*n* = 19)	Non-GCh Patients (*n* = 17)
Mean age in years (SD)	60.8 (13.1)	60.1 (13.3)
Sex (M:F)	9:11	5:15
Mean BMI (SD)	27.2 (5.6)	27.5 (4.0)
Mean Cigarettes Smoked/day (SD)	0 (0)	0 (0)
Mean Alcohol Units/week (SD)	4.3 (4.2)	4.14 (3.8)

**Table 3:** Sparse logistic regression analysis of FAIMS data from GCh and non-GCh (control) subjects.

		FAIMS Sparse Logistic Regression 100 Features					
		AUC (±95% CI)	Sensitivity (±95% CI)	Specificity (±95% CI)	PPV	NPV	*p* Values
GCh	Day 0						
	Day 7	0.92 (0.85–0.99)	0.75 (0.51–0.91)	0.89 (0.69–0.99)	0.88	0.77	0.000002
	Day 14	0.80 (0.64–0.95)	0.80 (0.56–0.94)	0.84 (0.60–0.97)	0.84	0.80	0.0006
	Day 28	0.71 (0.53–0.89)	0.94 (0.71–0.99)	0.47 (0.24–0.71)	0.61	0.90	0.014
	Day 56	0.77 (0.62–0.93)	0.73 (0.44–0.92)	0.68 (0.43–0.87)	0.65	0.76	0.003
Non-GCh	Day 0						
	Day 7	0.79 (0.63–0.95)	0.69 (0.41–0.89)	0.86 (0.57–0.98)	0.85	0.71	0.002
	Day 14	0.40 (0.18–0.61)	0.56 (0.30–0.80)	0.57 (0.29–0.82)	0.60	0.53	0.84
	Day 28	0.52 (0.29–0.76)	1 (0.71–1)	0.21 (0.05–0.51)	0.50	1	0.52
	Day 56	0.58 (0.34–0.83)	0.27 (0.06–0.61)	1 (0.77–1)	1	0.64	0.25

**Table 4:** Gaussian process analysis of GC-TOF-MS data from GCh and non-GCh (control) subjects.

		GC-TOF-MS					
		AUC (±95% CI)	Sensitivity (±95% CI)	Specificity (±95% CI)	PPV	NPV	*p* Values
GCh patients	Day 0						
	Day 7	0.75 (0.59–0.89)	0.65 (0.44–0.83)	0.64 (0.42–0.85)	0.69	0.60	0.02
	Day 14	0.71 (0.53–0.87)	0.71 (0.52–0.88)	0.69 (0.45–0.90)	0.75	0.64	0.05
	Day 28	0.55 (0.38–0.74)	0.65 (0.44–0.84)	0.54 (0.31–0.78)	0.65	0.54	0.63
	Day 56	0.51 (0.31–0.70)	0.71 (0.50–0.88)	0.54 (0.30–0.75)	0.67	0.58	0.93
Non-GCh patients	Day 0						
	Day 7	0.48 (0.27–0.69)	0.62 (0.38–0.83)	0.22 (0.00–0.50)	0.53	0.29	0.89
	Day 14	0.44 (0.25–0.65)	0.38 (0.15–0.62)	0.38 (0.17–0.62)	0.38	0.38	0.70
	Day 28	0.46 (0.26–0.67)	0.62 (0.38–0.83)	0.33 (0.10–0.60)	0.57	0.38	0.76
	Day 56	0.53 (0.31–0.74)	0.62 (0.38–0.83)	0.33 (0.10–0.60)	0.57	0.38	0.84

**Table 5:** Serum TTG levels for GCh and non-GCh patients.

	Day 0	Day 14	Day 56
GCh patients Mean TTG (U/mL) (SD)	2.43 (3.11)	3.60 (5.59)	3.58 (5.41)
Control Cohort Mean TTG (U/mL) (SD)	1.17 (0.38)	1.29 (0.59)	1.10 (0.28)

## Data Availability

Data available from the authors on request.
